# Young people’s attitudes towards wife-beating: Analysis of the Ghana demographic and health survey 2014

**DOI:** 10.1371/journal.pone.0245881

**Published:** 2021-02-02

**Authors:** Emmanuel Anongeba Anaba, Adom Manu, Deda Ogum-Alangea, Emefa Judith Modey, Adolphina Addo-Lartey, Kwasi Torpey

**Affiliations:** 1 Department of Population, Family and Reproductive Health, School of Public Health, University of Ghana, Accra, Ghana; 2 Department of Epidemiology and Disease Control, School of Public Health, University of Ghana, Accra, Ghana; University of Michigan, UNITED STATES

## Abstract

**Introduction:**

Intimate Partner Violence is a global public health problem. Attitude towards wife-beating is a major determinant of both intimate partner violence perpetration and victimization. However, little is known about the attitudes of Ghanaian young people towards wife-beating. The objectives of this study were to assess young people’s attitudes towards wife-beating, and identify salient factors influencing young people’s acceptance of wife-beating.

**Methods:**

Data used in this study were obtained from the 2014 Ghana Demographic and Health Survey. The survey was nationally representative and provides estimates for population and health indicators across the former ten regions of Ghana, including rural and urban areas. Data were analyzed with Stata/SE version 16.

**Results:**

We found that 32% of young women and 19% of young men accepted wife-beating. Among young women, acceptance of wife-beating was significantly influenced by younger age, wealth index, low educational status, religion, the region of residence, ethnicity, frequency of reading newspaper and frequency of listening to radio (*p* < 0.05). Among young men, acceptance of wife-beating was significantly influenced by wealth index, the region of residence and frequency of reading newspaper (*p* < 0.05).

**Conclusion:**

This study demonstrates that a substantial proportion of young people in Ghana accept wife-beating. Young women were more likely to accept wife-beating compared to young men. Acceptance of wife-beating was influenced by socio-demographic and behavioral factors. Efforts to end violence against women and girls in Ghana should focus on promoting girl education, economic empowerment of women and public education on laws that prohibit Intimate Partner Violence.

## Introduction

Intimate Partner Violence (IPV) is a global public health problem and a violation of human rights. Global calls to end IPV include the United Nations Declaration on Elimination of Violence Against Women [1]. Also, ending IPV is a major target for the fifth United Nations Sustainable Development Goal (Achieve gender equality and empower all women and girls) [2]. IPV refers to any act of physical aggression, sexual coercion, psychological or emotional abuse, or controlling behaviours by a current or former partner or spouse [1,3]. It is associated with physical, psychosocial, mental, sexual and reproductive health risks [4,5]. For instance, IPV is associated with unwanted pregnancy, injury, depression among others [1,6]. Globally, one in three women experiences IPV [7]. Wife-beating is a form of IPV against women, which is more prevalent in low-and middle-income countries (LMICs) [8,9].

In Ghana, IPV is a major public health problem. Evidence show that 40% of women who have ever married experienced physical violence, 35% experienced sexual violence and 58% experienced psychological violence [10]. To eliminate IPV, the Government of Ghana passed the Domestic Violence Act (Act 732) in 2007 [11]. The Ghana Police Service also operates the Domestic Violence and Victim Support Unit, which offers support services to victims of domestic violence, especially women [12].

Although there are multiple risk factors of IPV, studies have shown that attitude towards wife-beating is a major determinant of both IPV perpetration and victimization [13–15]. For instance, people who believe that IPV is acceptable are more likely to perpetrate it or be victimized [16]. Young people (persons between the ages of 10–24 years) are more likely to accept wife-beating, and mostly acquire the attitude from older generations, such as parents, through social learning [17–21]. Evidence suggest that men who were abused during childhood or witnessed paternal abuse against their mothers are more likely to accept wife-beating or become wife beaters [19,21]. Besides, young people are more vulnerable to IPV perpetration and victimization [18,19]. Therefore, understanding young people’s attitudes towards wife-beating are crucial in the fight against IPV. This will greatly inform the design of effective programmes and interventions by stakeholders [22–24].

According to the 2010 Population and Housing Census, young people constitute 31.8% of Ghana’s population [25]. A prior study in Ghana found that young people (15–24 years) were more likely to support wife-beating compared to adults (35–49 years) [26]. Nonetheless, little is known about predictors of young people’s attitudes towards IPV. This study, therefore, sought to assess young people’s attitudes towards wife-beating, analyzing the 2014 Ghana Demographic and Health Survey (GDHS) data. Specifically, we sought to assess young people’s attitudes towards wife-beating, and identify salient factors influencing young people’s acceptance of wife-beating. We hypothesized that adolescents, young people with no formal education and those from poor households were more likely to accept wife-beating.

## Methods

### Data and study design

Data used in this study were obtained from the 2014 GDHS [27]. The survey was nationally representative and provided estimates for population and health indicators across the former ten regions of Ghana, including rural and urban areas. The 2014 GDHS used a list of enumeration areas constructed for the 2010 Population and Housing Census as the sample frame. The 2014 GDHS utilized a two-stage stratified sampling design with probability proportional to size. Firstly, 427 enumeration areas comprising 216 rural and 211 urban areas, were selected using probability proportional to size. Secondly, 12,810 households were selected using a systematic sampling technique. All women aged 15–49 years who were usual members of the selected households or who spent the night before the survey in the selected households were eligible for the female survey. In half of the selected households, all men aged 15–49 years who were usual members of the households or who spent the night before the survey in the selected households were eligible for the male survey. In all, 3,150 young women and 1,423 young men between the ages of 15–24 years were interviewed. Written consent was obtained from young people above 18 years, those in a union, and parents of adolescents who were less than 18 years. Assent was obtained from adolescents who were less than 18 years. The 2014 GDHS was reviewed and approved by the Ghana Health Service Ethics Review Committee. Further details about the 2014 GDHS are provided elsewhere [27].

### Measures

The main outcome in this study was justified wife-beating. This was measured as a composite variable comprising five related items that sought to assess respondents’ acceptance of wife-beating. All the items were coded (0 = ‘No’ and 1 = ‘Yes’). The items include wife-beating justified: (i) if the wife goes out without telling her husband; (ii) if the wife neglects her children; (iii) if the wife argues with her husband; (iv) if the wife refuses to have sex with her husband; and (v) if the wife burns food. The scale had a relatively high internal consistency, with a reliability coefficient of 0.8. We then computed a composite index for the outcome variable by adding up the five items. We created a dummy outcome by categorizing the composite index scores. An index score of zero (0) for all the five items was labeled ‘wife-beating not justified’ and an index score of 1–5 was labeled ‘wife-beating justified’.

The exposure variables identified in the literature were age, education, marital status, wealth index, religion, residential status, ethnicity and region. Some behavioral factors including frequency of reading newspaper/magazine, frequency of listening to radio and frequency of watching television were also identified in the literature [26,28,29]. For clarity purposes, some of the variables were recoded. These include religion, which was recoded into four categories (1 = Christianity, 2 = Islam, 3 = Traditional and 4 = no religion). Similarly, ethnicity was recoded into five categories including (1 = Akan, 2 = Ewe, 3 = Mole-dagbani, 4 = Gurma and 5 = others (i.e. Grusi, Guan). Since the focus of this study was on young people (15–24 years), age groups outside this bracket were dropped from the dataset. We recoded age into (1 = adolescent (15–19 years); and 2 = young adult (20–24 years)). Marital status was recoded as (1 = single and 2 = married/cohabitating). Educational status was coded as 0 = no education, 1 = primary, 2 = secondary, 3 = tertiary. Wealth index was coded as 1 = poorest, 2 = poorer, 3 = middle, 4 = rich, 5 = richest, which was based on household income, expenditure and asset. Residential status was coded as 1 = urban, 2 = rural, while region was coded as 1 = Western, 2 = Central, 3 = Greater Accra, 4 = Volta, 5 = Eastern, 6 = Ashanti, 7 = Brong- Ahafo, 8 = Northern, 9 = Upper East and 10 = Upper West. The following behavioral variables (frequency of listening to radio, watching television and reading newspaper/magazine) were coded as 0 = not at all, 1 = less than once a week, 2 = at least once a week.

### Statistical analysis

Data were analyzed with Stata/SE version 16 (StataCorp, College Station, Texas, USA). Statistical analysis in this study was done using univariate and multivariable analyses. In the univariate analysis, we computed descriptive statistics, including frequency, percentage, mean and standard deviation. In the multivariable analysis, logistic regression analysis was conducted to identify predictors of the outcome variable. Two regression models were constructed, model 1 for young men and model 2 for young women. The models assessed the effect of the combined effort of socio-demographic variables and behavioral factors on the outcome variable. The analysis took into consideration the complex survey design. To off-set challenges associated with oversampling, survey weight was applied. A hierarchical backward variable selection approach was employed to select variables into the final model. The highest bivariate correlation coefficient between the predictors was 0.6, which is less than the recommended ≥ 0.7 [30], hence, there was no problem of collinearity. Furthermore, the Hosmer-Lemeshow test was employed to check the fitness of the models. All the models fitted reasonably well (p > 0.05) [31].

## Results

### Descriptive statistics

Among the 3,150 young women, half (50.7%) were between the ages of 20–24 years, with a mean age of 19 years and a standard deviation of 2.9. For the 1,423 young men, more than half (58.7%) were between the ages of 15–19 years, with a mean age of 19 years and a standard deviation of 2.8. Majority of the respondents had attained secondary education (young women = 69.8% and young men = 76.6%). Exactly 23.1% of young men and 22.1% of young women were in the richer wealth quintile. Also, majority (80.2% of young women and 75.9% of young men) of the respondents were Christians. A little above half of the respondents, young women (51.3%) and young men (51.1%), resided in urban areas. Also, 73.4% of young women and 64.3% of young men did not read newspaper at all. Regarding listening to radio, 49.7% of young women and 73.4% of young men reported listening to radio at least once a week. Last but not least, 52.9% and 64.2% of young women and young men watch television at least once a week respectively. See Table 1.

**Table 1:** Socio-demographic characteristics of respondents.

Characteristics	Young women	Young men
	n (%)	n (%)
**Age**		
Adolescents (15–19 years)	1553(49.3)	835(58.7)
Young adult (20–24 years)	1597(50.7)	588(41.3)
**Education status**		
No formal education	254(8.1)	46(3.3)
Primary education	568(18)	228(16)
Secondary education	2200(69.8)	1090(76.6)
Tertiary education	128(4.1)	56(4.1)
**Marital status**		
Single	2474(76.1)	1404(95.1)
Married/cohabitating	777(23.9)	72(4.9)
**Wealth index**		
Poorest	548(17.4)	281(19.7)
Poorer	602(19.1)	268(18.8)
Middle	684(21.7)	294(20.7)
Richer	697(22.1)	329(23.1)
Richest	619(19.7)	251(17.7)
**Religion**		
Christianity	2527(80.2)	1079(75.9)
Islam	511(16.2)	249(17.5)
Traditional	41(1.3)	29(2)
No religion	71(2.3)	66(4.6)
**Type of residence**		
Urban	1617(51.3)	728(51.1)
Rural	1533(48.7)	695(48.9)
**Region**		
Western	366(11.6)	165(11.6)
Central	299(9.5)	129(9.1)
Greater Accra	568(18.1)	271(19.1)
Volta	237(7.5)	114(8)
Eastern	309(9.8)	156(10.9)
Ashanti	578(18.3)	251(17.7)
Brong-Ahafo	296(9.4)	126(8.8)
Northern	271(8.6)	110(7.7)
Upper East	142(4.5)	64(4.5)
Upper West	85(2.7)	37(2.6)
**Ethnicity**		
Akan	1540(48.9)	729(51.3)
Ewe	406(12.9)	235(16.5)
Mole-Dagbani	506(16)	185(13)
Gurma	213(6.8)	187(13.1)
Other (i.e. Grusi, Guan)	486(15.4)	87(6.1)
**Frequency of reading newspaper/magazine**		
Not at all	2310(73.4)	915(64.3)
Less than once a week	480(15.3)	274(19.2)
At least once a week	357(11.4)	234(16.5)
**Frequency of listening to radio**		
Not at all	549(17.4)	113(7.9)
Less than once a week	1036(32.9)	266(18.7)
At least once a week	1565(49.7)	1044(73.4)
**Frequency of watching television**		
Not at all	662(21)	229(16.1)
Less than once a week	821(26.1)	281(19.7)
At least once a week	1667(52.9)	913(64.2)

### Attitudes towards wife-beating among young people

The findings showed that 12% of young women and 10% of young men justified wife-beating, if the wife goes out without telling her husband. It was also found that 24% of young women and 12% of young men justified wife-beating, if the wife neglects her children. Furthermore, 9% and 17% of young men and young women respectively, justified wife-beating, if the wife argues with her husband. Regarding the wife refusing to have sex with her husband, 12% of young women and 7% of young men justified wife-beating. Last but not least, 9% of young women and 5% of young men justified wife-beating, if the wife burns food. Overall, 32% of young women and 19 of young men justified wife-beating under all the five scenarios. See Table 2.

**Table 2:** Justification of wife-beating.

Characteristic		Young women	Young men
	Response	n (%)	n (%)
Beating justified; if the wife goes out without telling her husband	No	2581(82)	1282(90)
	Yes	569(18)	141(10)
Beating justified; if the wife neglects her children	No	2409(76)	1255(88)
	Yes	741(24)	168(12)
Beating justified; if the wife argues with her husband	No	2621(83)	1293(91)
	Yes	529(17)	130(9)
Beating justified; if the wife refuses to have sex with her husband	No	2762(88)	1322(93)
	Yes	388(12)	101(7)
Beating justified; if the wife burns food	No	2871(91)	1350(95)
	Yes		
Total score (index)	No	2151(68)	1157(81)
	Yes	999(32)	266(19)

### Logistic regression analysis of predictors of justified wife-beating

Model 1 (young men) focused on predictors of justified wife-beating among young men, while model 2 (young women) focused on predictors of justified wife-beating among young women. In model 1 (young men), we found that education, wealth index, region, religion and frequency of reading newspaper were significant predictors of wife-beating justified, if the wife goes out without telling her husband (p < 0.05). In model 2 (young women), we found that education, wealth index, region, religion and marital status were significant predictors of wife-beating justified, if the wife goes out without telling her husband (p < 0.05). Region also significantly predicted wife-beating justified, if the wife neglects her children in model 1 (young men), while, age, education, wealth index, religion and frequency of reading newspaper were significant predictors in model 2 (young women) (p < 0.05). In model 1 (young men), marital status, region, religion, ethnicity and frequency of reading newspaper were significant predictors of wife-beating justified, if the wife argues with her husband (p < 0.05). In model 2 (young women), education and region were found as significant predictors of wife-beating justified, if the wife argues with her husband (p < 0.05). Concerning wife-beating justified, if the wife refuses to have sex with her husband, ethnicity and frequency of reading newspaper emerged as significant predictors in model 1 (young men), while, education, region, ethnicity and frequency of reading newspaper emerged as significant predictors in model 2 (young women) (p < 0.05). While religion, region and marital status were significant predictors of wife-beating justified, if the wife burns food in model 1 (young men), education, wealth index, region, ethnicity and frequency of reading newspaper were significant predictors in model 2 (young women) (p < 0.05).

Regarding the overall attitude towards justification of wife-beating, we found that young women between the ages of 20–24 years had an 18% of reduced odds of justifying wife-beating compared to young women between the ages of 15–19 years (AOR = 0.82; CI = 0.69–0.96). It was also found that young women who had secondary education had 44% of reduced odds of justifying wife-beating compared to young women with no formal education (AOR = 0.56; CI = 0.42–0.75). Moreover, young women who had tertiary education had 84% of reduced odds of justifying wife-beating compared to young women with no formal education (AOR = 0.16; CI = 0.07–0.37). We also found that young women in the richer wealth quintile had 32% of reduced odds of justifying wife-beating compared to young women in the poorest wealth quintile (AOR = 0.68; CI = 0.47–0.96). Similarly, young women in the richest wealth quintile had 46% of reduced odds of justifying wife-beating compared to young women in the poorest wealth quintile (AOR = 0.54; CI = 0.36–0.81). On the other hand, young men in the richer wealth quintile had 54% reduced odds of justifying wife-beating compared to young men in the poorest wealth quintile (AOR = 0.46; CI = 0.25–0.85). Also, young men in the richest wealth quintile had 55% of reduced odds of justifying wife-beating compared to young men in the poorest wealth quintile (AOR = 0.45; CI = 0.22–0.92).

Besides, we found that young women in the Brong-Ahafo region were 1.6 times more likely to justify wife-beating compared to young women in the Western region (AOR = 1.63; CI = 1.21–2.32). Young women professing Islam were 1.5 times more likely to justify wife-beating compared to young women professing Christianity (AOR = 1.49; CI = 1.19–1.87). Regarding behavioral factors, young women who read newspaper at least once a week had 42% of reduced odds of justifying wife-beating compared to young women who did not read newspaper at all (AOR = 0.58; CI = 0.42–0.79). We also found that young women who listen to radio less than once a week had 34% of reduced odds of justifying wife-beating compared to young women who did not listen to radio at all (AOR = 0.66; CI = 0.52–0.33). Young women who listen to radio at least once a week had 28% of reduced odds of justifying wife-beating compared to young women who did not listen to radio at all (AOR = 0.72; CI = 0.58–0.89). See Tables 3 and 4.

**Table 3:** Logistic regression of predictors of justified wife-beating among young men in Ghana, 2014.

Model 1 (Young men)	Wife goes out without telling her husband	Wife neglect her children	Wife argues with her husband	Wife refuses to have sex with her husband	Wife burns food	Total (index score)
**Age**						
Adolescent (15–19 years)	1(ref)	1(ref)	1(ref)	1(ref)	1(ref)	1(ref)
Young adult (20–24 years)	0.89(0.59–1.36)	0.99(0.68–1.45)	1.13(0.73–1.75)	1.01(0.62–1.68)	0.98(0.55–1.95)	1.13(0.83–1.49)
**Education**						
None	1(ref)	1(ref)	1(ref)	1(ref)	1(ref)	1(ref)
Primary	0.45(0.18–1.14)	1.79(0.69–4.75)	1.30(0.43–3.72)	0.78(0.28–2.21)	2.43(0.94–6.26)	0.80(0.41–1.59)
Secondary	0.43(0.19–0.98) *	1.19(0.48–2.99)	0.67(0.25–1.82)	0.54(0.21–1.41)	2.21(0.87–5.67)	0.73(0.38–1.39)
Tertiary	0.99(0.24–4.24)	0.81(0.13–5.17)	0.79(0.12–5.28)	2.14(0.34–13.65)	3.78(0.36–39.85)	0.40(0.12–1.31)
**Marital status**						
Single	1(ref)	1(ref)	1(ref)	1(ref)	1(ref)	1(ref)
Married	0.50(0.18–1.37)	0.62(0.26–1.50)	0.33(0.11–0.99) *	0.65(0.22–1.93)	0.14(0.03–0.62) *	0.59(0.29–1.17)
**Wealth index**						
Poorest	1(ref)	1(ref)	1(ref)	1(ref)	1(ref)	1(ref)
Poorer	0.78(0.41–1.46)	0.88(0.42–1.84)	1.22(0.61–2.43)	0.79(0.38–1.61)	0.78(0.27–2.23)	0.77(0.49–1.20)
Middle	0.74(0.35–1.55)	0.78(0.38–1.63)	1.15(0.57–2.32)	0.76(0.32–1.83)	1.12(0.35–2.95)	0.71(0.43–1.16)
Richer	0.39(0.16–1.01) *	0.69(0.29–1.66)	0.88(0.34–2.15)	0.86(0.30–2.49)	0.73(0.15–3.42)	0.46(0.25–0.85) *
Richest	0.27(0.09–0.85) *	0.63(0.21–1.87)	0.46(0.16–1.33)	0.37(0.09–1.59)	0.28(0.05–1.73)	0.45(0.22–0.92) *
**Type of residence**						
Urban	1(ref)	1(ref)	1(ref)	1(ref)	1(ref)	1(ref)
Rural	0.86(0.49–1.49)	0.93(0.56–1.57)	1.29(0.79–2.12)	1.03(0.53–1.99)	1.19(0.56–2.55)	0.99(0.69–1.43)
**Region**						
Western	1(ref)	1(ref)	1(ref)	1(ref)	1(ref)	1(ref)
Central	0.58(0.23–1.49)	0.47(0.21–1.01) *	0.96(0.41–2.12)	1.64(0.62–4.33)	0.32(0.10–1.02)	1.01(0.59–1.74)
Greater Accra	1.21(0.46–2.75)	0.40(0.19–0.85) *	0.43(0.16–1.11)	1.38(0.49–3.83)	0.50(0.14–1.81)	0.80(0.43–1.49)
Volta	0.50(0.14–1.83)	0.16(0.05–0.53) *	0.33(0.11–1.03)	0.14(0.02–1.33)	0.21(0.04–1.22)	0.38(0.16–0.92) *
Eastern	0.73(0.33–1.62)	0.60(0.31–1.15)	1.20(0.53–0.96)	1.39(0.45–4.33)	0.49(0.16–1.51)	0.96(0.56–1.64)
Ashanti	0.54(0.24–1.24)	0.30(0.13–0.69) *	0.55(0.18–1.66)	1.31(0.37–3.48)	0.73(0.26–2.09)	0.45(0.23–0.86) *
Brong-Ahafo	0.35(0.15–0.83) *	0.19(0.08–0.48)	0.39(0.16–0.96) *	0.91(0.31–2.68)	0.19(0.06–0.32) *	0.29(0.16–0.55) *
Northern	0.49(0.15–1.67)	0.41(0.14–1.56)	1.43(0.50–4.09)	1.96(0.67–5.71)	0.33(0.09–1.21)	0.48(0.23–1.00)
Upper East	0.31(0.09–1.05)	0.26(0.09–0.69)	0.25(0.06–0.96) *	0.69(0.18–2.73)	0.32(0.08–1.32)	0.38(0.18–0.78) *
Upper West	0.63(0.19–2.11)	0.78(0.29–2.11)	1.93(0.69–5.38)	2.75(0.84–9.04)	0.65(0.18–2.39)	1.46(0.74–2.87)
**Religion**						
Christianity	1(ref)	1(ref)	1(ref)	1(ref)	1(ref)	1(ref)
Islam	1.39(0.84–2.31)	0.94(0.49–1.38)	1.46(0.69–3.04)	1.76(0.94–3.28)	1.21(0.44–3.34)	0.82(0.55–1.22)
Traditional	1.45(0.46–4.53)	1.61(0.76–3.31)	0.98(0.23–4.23)	0.83(0.19–3.66)	1.78(0.56–5.56)	1.18(0.57–2.42)
No religion	2.44(1.22–4.85) *	2.47(1.19–5.09)	2.72(1.19–6.25) *	1.03(0.28–3.74)	2.79(1.02–7.63) *	1.68(0.96–2.92)
**Ethnicity**						
Akan	1(ref)	1(ref)	1(ref)	1(ref)	1(ref)	1(ref)
Ewe	0.34(0.13–0.89) *	0.64(0.28–1.47)	1.12(0.47–2.63)	0.57(0.13–2.42)	1.69(0.48–6.02)	0.57(0.28–1.07)
Mole-Dagbani	0.56(0.23–1.34)	0.72(0.33–1.58)	0.31(0.14–0.67) *	0.33(0.13–0.79) *	0.79(0.19–3.19)	0.67(0.38–1.16)
Gurma	1.67(0.65–4.31)	1,98(0.86–4.57)	1.19(0.44–3.19)	1.34(0.50–3.66)	0.76(0.17–2.90)	1.28(0.68–2.42)
Other (i.e. Grusi, Guan)	1.20(0.65–2.21)	1.43(0.75–2.74)	1.52(0.66–3.43)	1.69(0.48–2.87)	1.76(0.48–6.46)	1.40(0.89–2.18)
**Frequency of reading newspaper**						
Not at all	1(ref)	1(ref)	1(ref)	1(ref)	1(ref)	1(ref)
Less than once a week	0.76(0.39–1.43)	0.97(0.57–1.07)	0.49(0.26–0.94) *	0.79(0.38–1.66)	0.56(0.22–1.44)	0.87(0.58–1.30)
At least once a week	0.31(0.13–0.74) *	0.59(0.19–0.81)	0.56(0.30–1.44)	0.19(0.06–0.59) *	0.25(0.05–1.22)	0.58(0.36–0.95) *
**Frequency of listening to radio**						
Not at all	1(ref)	1(ref)	1(ref)	1(ref)	1(ref)	1(ref)
Less than once a week	0.99(0.43–2.28)	0.72(0.29–1.71)	0.76(0.34–1.71)	0.77(0.32–1.85)	1.17(0.48–2.88)	0.98(0.57–1.69)
At least once a week	0.73(0.33–1.59)	0.53(0.24–1.18)	0.66(0.30–1.44)	0.48(0.23–1.01)	0.77(0.35–1.69)	0.72(0.44–1.19)
**Frequency of watching television**						
Not at all	1(ref)	1(ref)	1(ref)	1(ref)	1(ref)	1(ref)
Less than once a week	0.73(0.41–1.29)	1.08(0.59–1.99)	1.56(0.83–2.93)	1.22(0.59–2.56)	0.90(0.44–1.85)	1.09(0.71–1.67)
At least once a week	0.79(0.45–1.41)	0.89(0.50–1.56)	1.26(0.69–2.29)	1.04(0.54–2.01)	0.71(0.39–1.29)	0.88(0.58–1.32)

Note: Ref (reference category)

*p -value < 0.05.

**Table 4:** Logistic regression of predictors of justified wife-beating among young women in Ghana, 2014.

Model 2 (Young women)	Wife goes out without telling her husband	Wife neglect her children	Wife argues with her husband	Wife refuses to have sex with her husband	Wife burns food	Total (index score)
**Age**						
Adolescent (15–19 years)	1(ref)	1(ref)	1(ref)	1(ref)	1(ref)	1(ref)
Young adult (20–24 years)	0.80(0.62–1.04)	0.71(0.56–0.91) *	0.83(0.64–1.07)	0.90(0.66–1.24)	0.83(0.61–1.14)	0.82(0.69–0.96) *
**Education**						
None	1(ref)	1(ref)	1(ref)	1(ref)	1(ref)	1(ref)
Primary	1.15(0.75–1.73)	0.76(0.50–1.16)	0.81(0.56–1.16)	0.59(0.38–0.92) *	0.82(0.54–1.24)	0.79(0.58–1.07)
Secondary	0.66(0.41–1.08)	0.48(0.32–0.72) *	0.43(0.29–0.64) *	0.30(0.19–0.47) *	0.52(0.32–0.83) *	0.56(0.42–0.75) *
Tertiary	0.19(0.42–0.88) *	0.17(0.06–0.51) *	0.12(0.02–0.63) *	0.16(0.03–0.73) *	0.24(0.03–2.02)	0.16(0.07–0.37) *
**Marital status**						
Single	1(ref)	1(ref)	1(ref)	1(ref)	1(ref)	1(ref)
Married	1.44(1.05–1.98) *	1.04(0.78–1.39)	1.22(0.92–1.62)	1.25(0.87–1.80)	1.07(0.74–1.54)	0.98(0.79–1.21)
**Wealth index**						
Poorest	1(ref)	1(ref)	1(ref)	1(ref)	1(ref)	1(ref)
Poorer	0.73(0.52–1.04)	0.97(0.72–1.31)	1.10(0.79–1.54)	0.88(0.55–1.39)	0.67(0.41–1.08)	0.91(0.70–1.17)
Middle	0.59(0.40–0.87) *	0.78(0.53–1.15)	1.03(0.69–1.53)	0.99(0.61–1.63)	0.48(0.28–0.84) *	0.75(0.56–1.01)
Richer	0.46(0.27–0.79) *	0.64(0.38–1.08)	0.77(0.44–1.36)	0.89(0.48–1.65)	0.44(0.22–0.87) *	0.68(0.47–0.96) v
Richest	0.35(0.18–0.67) *	0.51(0.29–0.91) *	0.57(0.28–1.14)	0.55(0.23–1.31)	0.38(0.14–1.03)	0.54(0.36–0.81) *
**Type of residence**						
Urban	1(ref)	1(ref)	1(ref)	1(ref)	1(ref)	1(ref)
Rural	1.14(0.77–1.67)	1.02(0.68–1.52)	1.12(0.74–1.69)	1.19(0.78–1.84)	0.94(0.57–1.55)	1.06(0.85–1.31)
**Region**						
Western	1(ref)	1(ref)	1(ref)	1(ref)	1(ref)	1(ref)
Central	0.61(0.28–1.31)	0.53(0.27–1.06)	0.49(0.21–1.14)	0.43(0.16–1.14)	0.59(0.23–1.54)	0.78(0.55–1.10)
Greater Accra	0.76(0.38–1.55)	0.69(0.35–1.35)	0.47(0.22–1.00) *	0.48(0.19–1.14)	0.48(0.18–1.29)	0.89(0.60–1.33)
Volta	0.56(0.25–1.27)	1.22(0.60–2.49)	0.62(0.26–1.48)	1.03(0.42–2.42)	1.19(0.47–3.04)	1.53(0.92–2.35)
Eastern	0.68(0.34–1.33)	0.64(0.36–1.14)	0.46(0.23–0.91) *	0.36(0.16–0.81) *	0.55(0.25–1.21)	0.90(0.63–1.29)
Ashanti	0.54(0.31–0.97) *	0.78(0.45–1.35)	0.61(0.33–1.10)	0.42(0.21–0.84) *	0.16(0.07–0.39) *	0.85(0.60–1.21)
Brong-Ahafo	1.06(0.61–1.88)	1.31(0.77–2.23)	0.95(0.52–1.77)	0.74(0.39–1.42)	0.51(0.27–0.94) *	1.63(1.21–2.32) *
Northern	0.79(0.41–1.52)	1.06(0.58–1.94)	0.71(0.36–1.42)	1.13(0.57–2.23)	0.82 (0.39–1.67)	1.43(0.90–2.08)
Upper East	0.49(0.26–0.92) *	0.74(0.42–1.29)	0.63(0.32–1.24)	0.42(0.22–0.84) *	0.45(0.22–0.89) *	0.72(0.48–1.09)
Upper West	0.44(0.22–0.89) *	0.62(0.29–1.32)	0.38(0.41–0.99) *	0.39(0.15–1.04)	0.43 (0.16–1.18)	1.02(0.66–1.52)
**Religion**						
Christianity	1(ref)	1(ref)	1(ref)	1(ref)	1(ref)	1(ref)
Islam	1.80(1.26–2.57) *	1.75(1.25–2.45) *	1.21(0.82–1.79)	1.27(0.86–1.87)	1.20(0.78–1.86)	1.49(1.19–1.87) *
Traditional	1.81(0.72–4.53)	2.59(0.96–7.04)	2.19(0.81–5.96)	2.13(0.83–5.45)	1.52(0.56–4.18)	1.56(0.26–3.18)
No religion	0.96(0.48–1.93)	0.91(0.46–1.80)	0.57(0.31–1.08)	0.93(0.43–1.99)	0.77(0.40–1.46)	1.02(0.58–1.74)
**Ethnicity**						
Akan	1(ref)	1(ref)	1(ref)	1(ref)	1(ref)	1(ref)
Ewe	0.85(0.46–1.56)	0.76(0.51–1.34)	0.86(0.47–1.58)	0.72(0.35–1.48)	0.76(0.34–1.68)	0.60(0.41–0.89) *
Mole-Dagbani	0.75(0.49–1.13)	0.62(0.37–1.03)	0.72(0.45–1.16)	1.24(0.79–1.95)	0.72(0.43–1.21)	0.79(0.59–1.06)
Gurma	1.89(1.21–2.96)	0.67(0.44–1.03)	2.39(1.40–4.09) *	2.26(1.28–3.99) *	2.11(1.16–3.84) *	1.73(1.17–2.54) *
Other (i.e. Grusi, Guan)	0.93(0.62–1.39)	1.55(0.93–2.59)	0.93(0.60–1.43)	1.02(0.65–1.62)	0.78(0.43–1.39)	0.86(0.66–1.31)
**Frequency of reading newspaper**						
Not at all	1(ref)	1(ref)	1(ref)	1(ref)	1(ref)	1(ref)
Less than once a week	0.71(0.49–1.05)	0.85(0.62–1.18)	0.84(0.58–1.21)	0.92(0.58–1.44)	0.51(0.31–0.86) *	0.94(0.73–1.19)
At least once a week	0.35(0.20–0.60)	0.46(0.28–0.97) *	0.36(0.91–0.66)	0.39(0.17–0.86) *	0.16(0.06–0.39) *	0.58(0.42–0.79) *
**Frequency of listening to radio**						
Not at all	1(ref)	1(ref)	1(ref)	1(ref)	1(ref)	1(ref)
Less than once a week	0.55(0.39–0.76)	0.66(0.48–0.89) *	0.76(0.54–1.08)	0.79(0.57–1.10)	0.89(0.59–1.36)	0.66(0.52–0.33) *
At least once a week	0.72(0.53–0.96)	0.84(0.63–1.11)	0.73(0.52–1.02)	0.94(0.67–1.32)	1.20(0.82–1.76)	0.72(0.58–0.89) *
**Frequency of watching television**						
Not at all	1(ref)	1(ref)	1(ref)	1(ref)	1(ref)	1(ref)
Less than once a week	0.96(0.69–1.36)	0.96(0.69–1.31)	1.01(0.72–1.39)	0.98(0.64–1.51)	0.85(0.51–1.42)	1.12(0.87–1.38)
At least once a week	1.09(0.79–1.50)	0.89(0.68–1.85)	1.09(0.79–1.51)	1.04(0.67–1.62)	1.14(0.73–1.76)	0.98(0.78–1.23)

Note: Ref (reference category)

*p -value < 0.05.

## Discussion

This study sought to assess young people’s attitudes towards wife-beating, and identify salient factors influencing young people’s acceptance of wife-beating. The findings showed that three in ten young women accepted wife-beating, while, about two in ten young men accepted wife-beating. These findings are supported by prior studies. For example, a study of 14 secondary schools in Jordan found that the prevalence of justified wife-beating ranged between 6.1% - 50.5% [17]. Ironically, we found that a higher proportion of young women accepted wife-beating compared to young men. This finding is contrary to what was found among adolescents in Armenia, where 30% of males and 24% of females supported wife-beating [32]. The differences in findings can be attributed to differences in contextual factors. Nonetheless, there is evidence to show that women in sub-Saharan Africa are more likely to support wife-beating [33]. For example, a study of seven sub-Saharan Africa countries revealed that about eight in ten women accepted wife-beating [34]. Possible reasons might be an economic vulnerability in combination with structural inequalities between men and women, a male-dominated society, marriage-related norms, and family structures [20]. This finding suggest that young women in Ghana are vulnerable to IPV victimization, since studies have shown that women who accept wife abuse are more likely to be abused by their partners [35,36]. In addition, young women who accept wife-beating may not seek help or report their partners if they are physically abused. Consequently, they may suffer silently in pain and psychological distresses. This finding sends a strong message to stakeholders who seek to promote the health and wellbeing of women and girls, such as the Ministry of Gender, Children and Social Protection. If the trend remains uninterrupted, it is probable that Ghana may not be able to achieve the fifth Sustainable Development Goal, which seeks to end violence against women and girls.

Our findings further showed that acceptance of wife-beating was influenced by age, region, religion, ethnicity, educational status, wealth index and access to information. Young women with no formal education and those from poor households were more likely to accept wife-beating. Similarly, young men from poor households were more likely to accept wife-beating. Hence, supporting the researchers’ hypotheses. These findings are parallel to existing studies across low-and-middle income-countries (LMICs) [15,37,38]. For instance, a study that analyzed nationally representative data of 39 LMICs found that respondents from Africa were more likely to accept wife-beating. The study also found that people of low socio-economic status, low formal education and younger age were more likely to accept wife-beating [18]. This implies that poverty, low education and younger age are risk factors of justified wife-beating. There is substantial evidence to show that these risk factors expose women to actual physical abuse [12,33,39]. In this regard, IPV prevention programmes or interventions should focus on more vulnerable populations, such as adolescents. Moreover, interventions aimed at promoting girl-child education and gender equality through economic empowerment initiatives are strongly recommended.

Our finding on the relationship between access to information and attitudes towards wife-beating is supported by earlier studies [37,38,40–42]. This implies that young people who listen to radio or read newspaper are more likely to be exposed to intimate partner violence prevention information. In this regard, the Ministry of Gender, Children and Social Protection should liaise with the National Commission on Civic Education to educate the public about laws against intimate partner violence via available media channels. Considering that the radio had positive effects on attitudes, stakeholders must utilize it together with other channels such as youth-oriented newspapers and print media to raise awareness of IPV. Stakeholders such as the Ministry of Gender, Children and Social Protection, the Domestic Violence and Victim Support Unit and allied institutions must work together to improve awareness and attitudes towards IPV. Stakeholders should adopt or adapt known and tested programmes such as the Safe Date and Shifting Boundaries, which have proven to be effective in reducing IPV perpetration and victimization among young people [43].

Schools present a golden opportunity for education and behavior improvement for in-school youth. The Ghana Education Service should work with appropriate agencies to incorporate elements of Gender-Based Violence and IPV prevention into existing and new curricular. Additionally, stakeholders should incorporate IPV prevention lessons into youth-friendly health programmes, including Comprehensive Sexuality Education. These strategies can help change young people’s attitudes towards IPV, as well as, reduce IPV perpetration and victimization. These interventions, however, must be youth-centered as well as encourage the participation of young people.

### Limitations

Although this study provides invaluable information for IPV prevention interventions in Ghana, it is not devoid of limitations. The researchers acknowledge that the cross-sectional nature of the study is a limitation. Therefore, the interpretation of the findings must be done with caution. It is recommended that future studies should adopt qualitative approaches to better expose the many intricate views of young people relating to issues of wife-beating beyond what quantitative studies show. Notwithstanding, the findings of this study underscore the risk factors of IPV. Stakeholders can leverage this empirical evidence to design interventions that can help eliminate violence against women and girls in Ghana.

## Conclusions

This study demonstrates that an appreciable proportion of young people in Ghana accept wife abuse. Acceptance of wife abuse is influenced by socio-demographic characteristics. Access to information is a protective factor against acceptance of wife abuse. Efforts to end violence against women and girls in Ghana should focus on promoting girl-child education, economic empowerment of women and public awareness and education about laws that prohibit IPV.
